# Supplementary figures and images for: Discovery of repurposing drug candidates for the treatment of diseases caused by pathogenic free-living amoebae
Source: PLoS Negl Trop Dis. 2020 Sep 24;14(9):e0008353. doi: 10.1371/journal.pntd.0008353 (PMC7546510; doi:10.1371/journal.pntd.0008353)

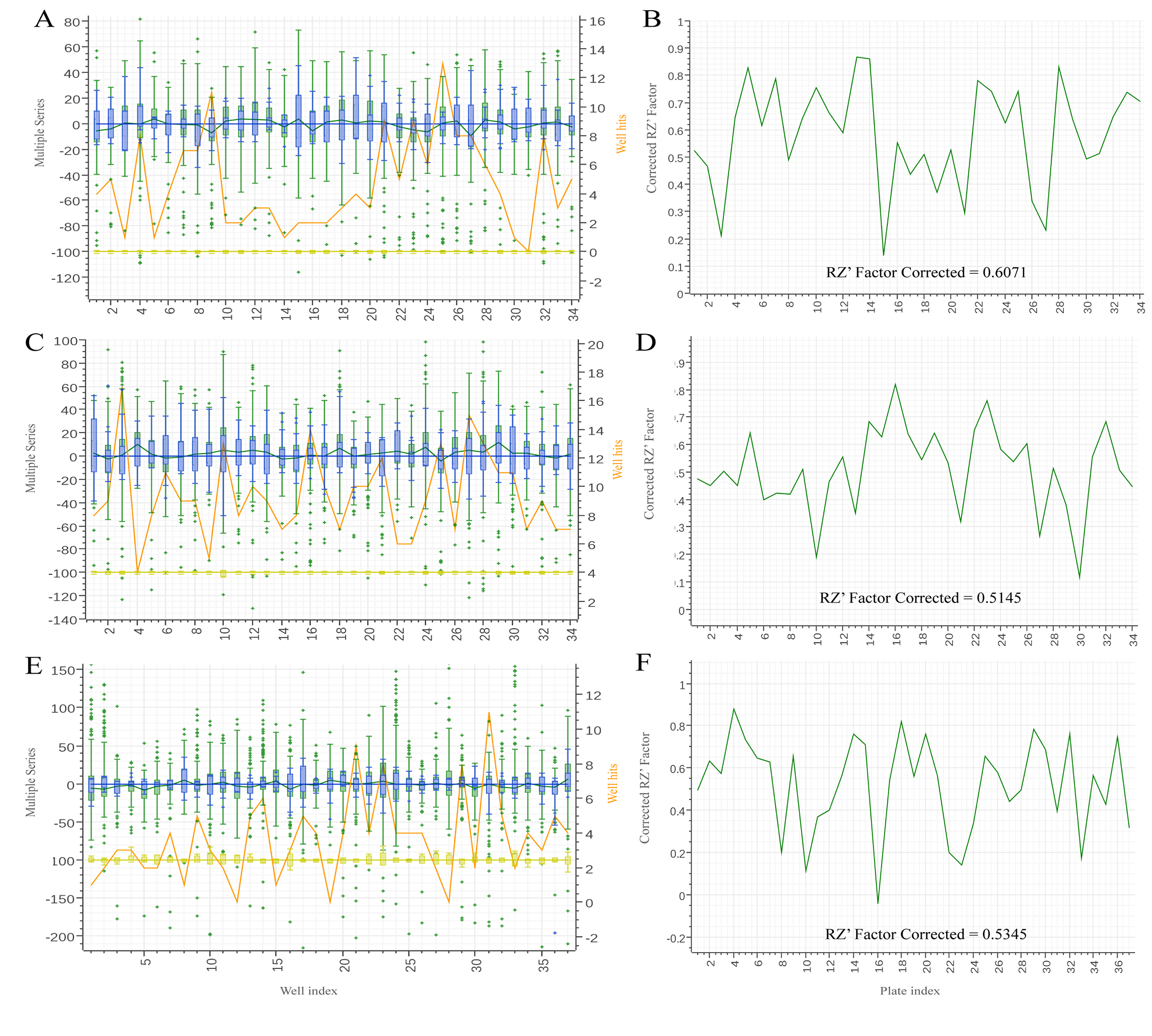

Supplement: S1 Fig — Plate statistics from screening the Calibr ReFRAME library at a single point of 5 μM against pathogenic N. fowleri (A & B), A. castellanii (C & D) and B. mandrillaris (E & F). In A, C, and E, each of the green bars represent the median compound response, the blue bars represent the median 0.5% DMSO (negative) control, the yellow bars represent the median drug controls (posaconazole for Naegleria and Acanthamoeba (A & C) and artovastatin for Balamuthia (E)). All plates were normalized to percent growth with the orange line representing the number of hits identified on each plate. We used the Z’ factor as a statistical measurement to assess the robustness of our high-throughput screening assays (B, D & F). This factor uses the mean and standard deviation values of the neutral controls minus the negative controls. The robust RZ’ factor median was found to be 0.6071, 0.5145, 0.5345 for N. fowleri (B), A. castellanii (D) and B. mandrillaris (F), respectively. (TIF) [file pntd.0008353.s001.tif]

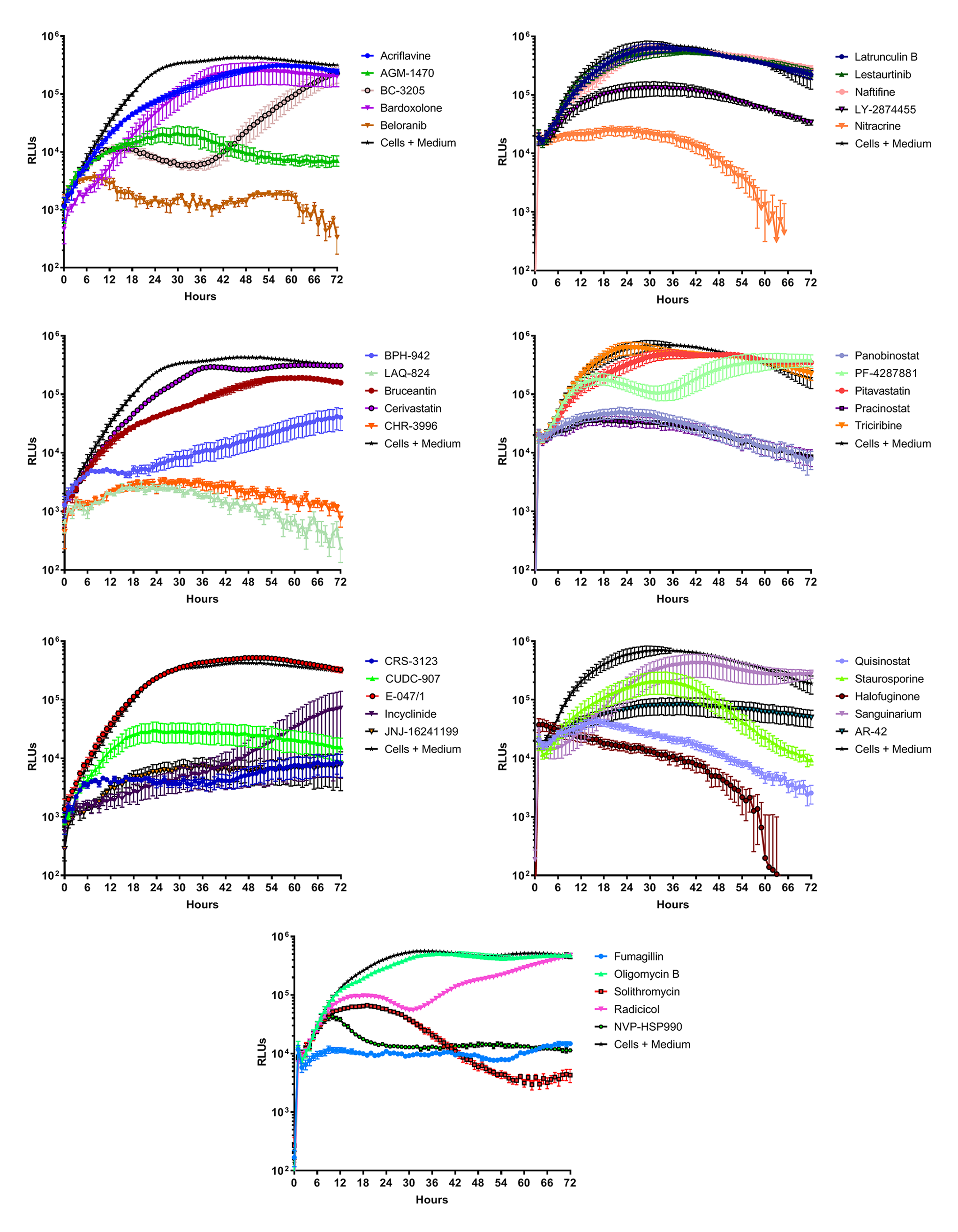

Supplement: S2 Fig — Rate of inhibition at the IC50 concentration for all drugs identified through the Calibr ReFRAME library screen on Naegleria fowleri. These graphs represent the pooled data from two independent biological experiments with 3 replicates (N = 2). (TIF) [file pntd.0008353.s002.tif]

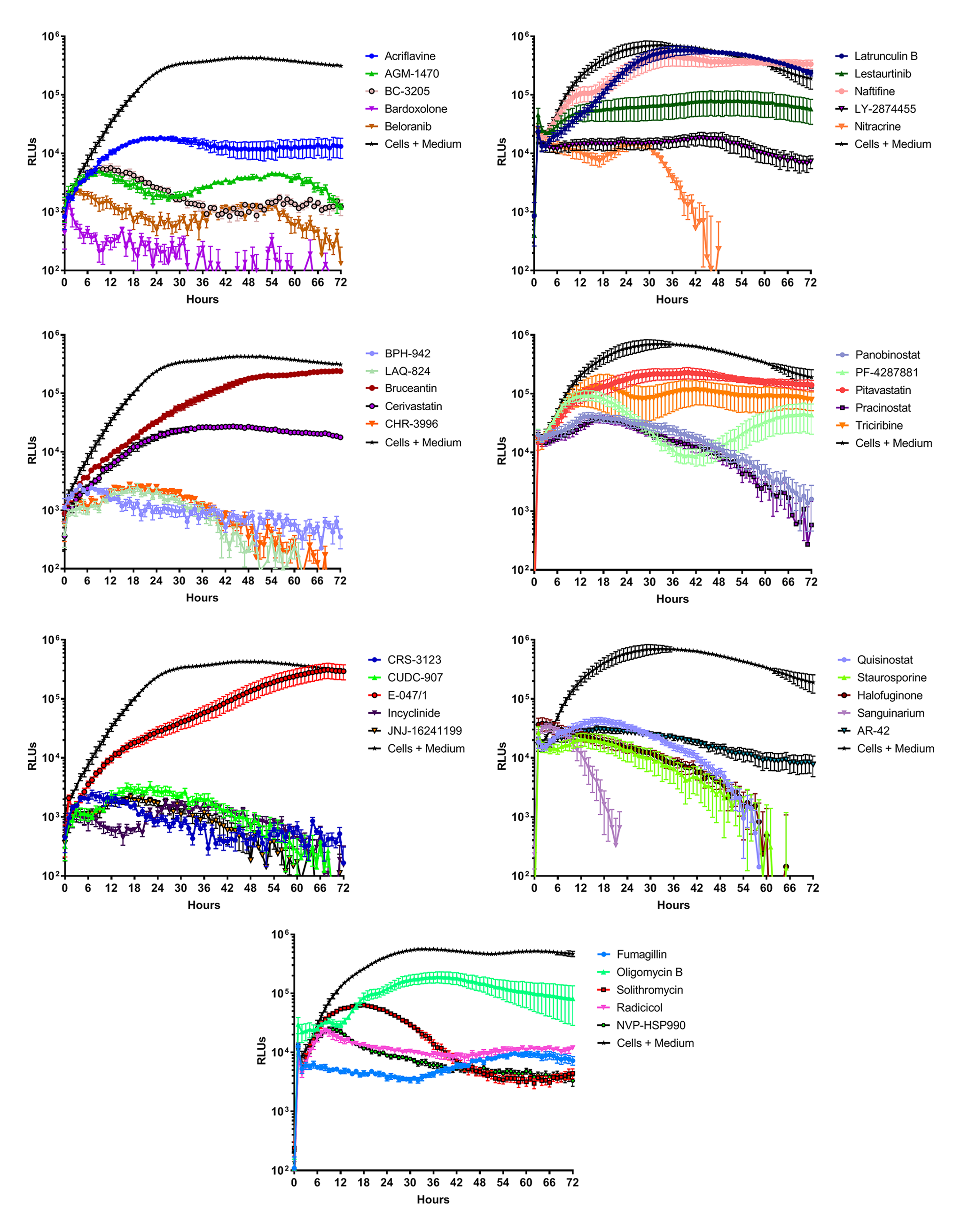

Supplement: S3 Fig — Rate of inhibition at 5x-IC50 concentration for all drugs identified through the Calibr ReFRAME library screen on Naegleria fowleri. These graphs represent the pooled data from two independent biological experiments with 3 replicates (N = 2). (TIF) [file pntd.0008353.s003.tif]
