# Supplementary material for: Discovery of repurposing drug candidates for the treatment of diseases caused by pathogenic free-living amoebae
Source: PLoS Negl Trop Dis. 2020 Sep 24;14(9):e0008353. doi: 10.1371/journal.pntd.0008353 (PMC7546510; doi:10.1371/journal.pntd.0008353)
Supplement: S1 Table — Hits identified in single point assays were selected for confirmation in quantitative dose-response assays. Each compound was run in two biological replicates and qAC50’s (μM; ± SE Log) determined. In addition, rate of action studies were conducted at 1x-IC50 concentration of drug. The potential mechanism of action of each hit was assessed from review of published literature. (PDF) [file pntd.0008353.s004.pdf]

S1 Table: *Naegleria fowleri* active compounds identified through dose-response (N=2)				
**Class**	**Compounds**	**qAC_{50}'s (μM) ± (SE Log)**	**Rate of Action at 1x-IC_{50} (Hours)**	**Proposed Mechanism of Action**
Antineoplastics	Beloranib hemioxalate	0.02 (0.06)	8 hours	Methionine Aminopeptidase-2 (MetAP2) inhibitor
	Fimepinostat	0.13 (0.04)	23 hours	Histone deacetylase (HDAC) inhibitor
	Fumagillin	0.15 (0.20)	10 hours	Methionine Aminopeptidase-2 (MetAP2) inhibitor
	AGM-1470	0.29 (0.11)	29 hours	Methionine Aminopeptidase-2 (MetAP2) inhibitor
	Latrunculin B	0.33 (0.06)	No effect	Disrupts actin cytoskeleton
	Bardoxolone	0.34 (0.03)	8 hours‡	NF-κB pathway inhibitor
	Bardoxolone methyl Δ	0.36 (0.011)	8 hours‡	NF-κB pathway inhibitor
	Nitracrine dihydrochloride	0.42 (0.02)	24 hours	Nucleoside inhibitor
	JNJ-16241199	0.52 (0.10)	36 hours	Histone deacetylase (HDAC) inhibitor
	Quisinostat	0.75 (0.12)	16 hours	Histone deacetylase (HDAC) inhibitor
	Tractinostat	0.97 (0.11)	26 hours	Histone deacetylase (HDAC) inhibitor
	AR-42	1.00 (0.04)	33 hours	Histone deacetylase (HDAC) inhibitor
	Panobinostat lactate	1.25 (0.25)	22 hours	Histone deacetylase (HDAC) inhibitor
	Lestaurtinib	2.31 (0.03)	No effect	Tyrosine kinase inhibitor
	NVP-HSP990	2.49 (0.17)	10 hours	HSP90 inhibitor
	LY-2874455	3.57 (0.19)	31 hours	Fibroblast growth factor inhibitor
	Dacinostat	3.83 (0.06)	20 hours	Histone deacetylase (HDAC) inhibitor
	Incyclinide	3.91 (0.05)	50 hours‡	Matrix metalloproteinases (MMPs) inhibitor
	Bruceantin	4.72 (0.11)	62 hours	MYC Expression inhibitor
	Pracinostat	5.31 (0.25)	17 hours	Histone deacetylase (HDAC) inhibitor
	PF-03814735	5.89 (0.03)	ND	Aurora Kinase inhibitor
	TG-02	6.06 (0.14)	ND	Cyclin-Dependent Kinase inhibitor
	Cenisertib	6.56 (0.14)	ND	Aurora Kinase inhibitor
	AZD-5438	6.58 (0.08)	ND	Aurora Kinase inhibitor

	Triciribine phosphate	6.68 (0.17)	No effect	Protein Kinase B (PKB/Akt) inhibitor
	Omipalisib	6.90 (0.21)	ND	mTOR Complex 1&2; Phosphatidylinositol 3-Kinase-α,-β,-γ,-δ inhibitor
Antibacterials	Oligomycin B	0.06 (0.03)	No effect	ATP synthase inhibitor
	Staurosporine	0.21 (0.04)	34 hours	Protein kinase C inhibitor
	Azithromycin ≠	0.29 (0.06)	26 hours	23S-rRNA of 50S ribosomal subunit inhibitor
	Valnemulin ≠	0.42 (0.22)	No effect	50S ribosomal subunit inhibitor
	Erythromycin ≠	0.77 (0.19)	ND	23S-rRNA of 50S ribosomal subunit inhibitor
	BC-3205	0.83 (0.11)	17 hours‡	23S-rRNA of 50S ribosomal subunit inhibitor
	CRS3123; REP3123	1.43 (0.11)	16 hours	Methionyl tRNA synthetase inhibitor
	Radicicol	11.8 (0.17)	13 hours‡	HSP90 inhibitor
	Roxithromycin ≠	3.37 (0.08)	No effect	23S-rRNA of 50S ribosomal subunit inhibitor
	Sanguinarium chloride	3.83 (0.13)	13 hours‡	Rho GTPase inhibitors
	Erythromycin stinoprate Δ	4.51 (0.03)	ND	23S-rRNA of 50S ribosomal subunit inhibitor
	Solithromycin	4.96 (0.13)	19 hours	23S-rRNA of 50S ribosomal subunit inhibitor
	PF-4287881	5.16 (0.20)	18 hours‡	50S ribosomal subunit inhibitor
	Clarithromycin ≠	6.03 (0.12)	ND	23S-rRNA of 50S ribosomal subunit inhibitor
	Mycaminosyl tylonolide	9.37 (0.12)	ND	50S ribosomal subunit inhibitor
Antifungals	Terbinafine hydrochloride ≠	0.23 (0.17)	36 hours	Squalene monoxygenase inhibitor
	Gentian violet	1.08 (0.07)	ND	Cyclin-Dependent Kinase 1B inhibitor
	Butenafine ≠	5.35 (0.12)	33 hours	Squalene monoxygenase inhibitor
	Naftifine hydrochloride	5.61 (0.23)	No effect	Squalene monoxygenase inhibitor
Antihyperlipidaemics	Cerivastatin sodium	1.09 (0.28)	41 hours‡	3-hydroxy-3-methyl-glutaryl-coenzyme A (HMG-CoA) inhibitor
	Pitavastatin calcium ≠	3.48 (0.17)	35 hours‡	3-hydroxy-3-methyl-glutaryl-coenzyme A (HMG-CoA) inhibitor
Antifibrotic	Halofuginone	1.49 (0.02)	2 hours	Prolyl tRNA synthetase inhibitor

Mycogenic toxin	Cytochalasin B	3.16 (0.09)	ND	Disrupts actin cytoskeleton
Diazo dye	Trypan Blue	3.58 (0.05)	ND	Selectively stains connective tissue
Antimalarial	BPH-942	5.1 (0.09)	11 hours‡	Farnesyl diphosphate synthase inhibitor
Antiseptic	Acriflavine	5.12 (0.18)	18 hours‡	PERK/eIF2a/ATF4 UPR pathway inhibitor
Class III Antiarrhythmic	E-04711	5.22 (0.04)	No effect	K^+ efflux/Na^+ channel antagonist

≠ Compounds we previously discovered through independent screening of an FDA approved compound library [49].

Δ Different salts included throughout the library.

‡ Indicates parasites recrudesced to untreated control values at the 72 hour time point.

SE - Standard Error, ND - Not Determined.
