# Supplementary material for: Discovery of repurposing drug candidates for the treatment of diseases caused by pathogenic free-living amoebae
Source: PLoS Negl Trop Dis. 2020 Sep 24;14(9):e0008353. doi: 10.1371/journal.pntd.0008353 (PMC7546510; doi:10.1371/journal.pntd.0008353)
Supplement: S2 Table — Hits identified in single point assays were selected for confirmation in quantitative dose-response assays. Each compound was run in two biological replicates and qAC50’s (μM; ± SE Log) determined. In addition, assays were conducted with 10 μM of each hit to assess if the compound has cysticidal, cystistatic or no activity against cysts. The potential mechanism of action of each hit was assessed from review of published literature. (PDF) [file pntd.0008353.s005.pdf]

S2 Table: *Acanthamoeba castellanii* active compounds identified through dose-response (N=2)				
**Class**	**Compounds**	**qAC_{50}'s (µM) ± (SE Log)**	**Cyst activity at 10 µM**	**Proposed Mechanism of Action**
Antifungals	Efinaconazole	0.03 (0.14)	No activity	Cell wall synthesis inhibitor
	Croconazole	0.03 (0.11)	No activity	Cell wall synthesis inhibitor; Lanosterol 14 α-demethylase inhibitor
	DuP-860	0.05 (0.07)	Cystistatic (day 11, recrudescence)	Cell wall synthesis inhibitor
	Aliconazole	0.21 (0.09)	No activity	Cell wall synthesis inhibitor
	Oteseconazole	0.25 (0.04)	Cystistatic (day 9, recrudescence)	Cell wall synthesis inhibitor; Lanosterol 14 α-demethylase inhibitor
	Climbazole	0.29 (0.04)	No activity	Cell wall synthesis inhibitor
	TAK 456	0.57 (0.14)	No activity	Cell wall synthesis inhibitor; Lanosterol 14 α-demethylase inhibitor
	Isavuconazole	0.85 (0.12)	No activity	Cell wall synthesis inhibitor; Lanosterol 14 α-demethylase inhibitor
	Thimerosol	1.26 (0.08)	No activity	Glutathione Transferase inhibitor
	T-23207	1.81 (0.02)	No activity	Disrupts mitochondrial function
	Becliconazole	2.47 (0.04)	No activity	Cell wall synthesis inhibitor
	Bifonazole	2.62 (0.07)	No activity	3-hydroxy-3-methyl-glutaryl-coenzyme A (HMG-CoA) inhibitor
	Voriconazole	2.70 (0.06)	No activity	Cell wall synthesis inhibitor; Lanosterol 14 α-demethylase inhibitor
	Butenafine	3.34 (0.13)	No activity	Squalene monoxygenase inhibitor
	Gentian violet	3.38 (0.13)	ND	Cyclin-Dependent Kinase 1B inhibitor
	Sulconazole nitrate	4.45 (0.05)	No activity	Cell wall synthesis inhibitor; Lanosterol 14 α-demethylase inhibitor
Antineoplastics	Pelitrexol	0.14 (0.13)	No activity	Glycinamide Ribonucleotide Formyltransferase (GARTFase) inhibitor
	5-fluoro-2'-deoxycytidine; FdCyd	0.19 (0.37)	No activity	DNA Methyltransferase (DNMT) inhibitor
	5-fluoro-2'-deoxyuridine; Floxuridine	0.43 (0.10)	No activity	Thymidylate synthase inhibitor
	2'-deoxy-2'-methylidenecytidine	0.85 (0.04)	No activity	DNA synthesis inhibitor
	Omipalisib	1.42 (0.14)	No activity	mTOR Complex 1&2; Phosphatidylinositol 3-Kinase-α,-β,-γ,-δ inhibitor

	FF-705	1.80 (0.07)	No activity	Thymidylate synthase inhibitor
Antiseptics	Olanexidine	1.97 (0.07)	No activity	Phospholipase inhibitor
	Chlorhexidine*	2.71 (0.04)	No activity	ATPase inhibitor; Membrane integrity inhibitor
	Alexidine*	2.73 (0.04)	No activity	Phospholipase inhibitor
	Decamethoxine	2.8 (0.12)	No activity	Phospholipase inhibitor
	Analog of Decamethoxine	3.57 (0.12)	No activity	Phospholipase inhibitor
Antimycin	Antimycin A	1.27 (0.12)	No activity	Cytochrome C reductase inhibitor
Antihyperglycaemic	LY-2881835	1.67 (0.09)	No activity	G-protein-coupled receptor 40 (GPR40) inhibitor
Antibacterial	Azithromycin	2.16 (0.18)	No activity	23S-rRNA of 50S ribosomal subunit inhibitor
Diazo dye	Trypan Blue	2.82 (0.05)	ND	Selectively stains connective tissue
Diamidine	Stilbamidine	2.91 (0.05)	No activity	Nucleoside inhibitor

* Compounds described to have cytsicidal activity at higher concentrations tested.

ND - Not Determined.
