# Supplementary material for: Discovery of repurposing drug candidates for the treatment of diseases caused by pathogenic free-living amoebae
Source: PLoS Negl Trop Dis. 2020 Sep 24;14(9):e0008353. doi: 10.1371/journal.pntd.0008353 (PMC7546510; doi:10.1371/journal.pntd.0008353)
Supplement: S3 Table — Hits identified in single point assays were selected for confirmation in quantitative dose-response assays. Each compound was run in two biological replicates and qAC50’s (μM; ± SE Log) determined. The potential mechanism of action of each hit was assessed from review of published literature. (PDF) [file pntd.0008353.s006.pdf]

S3 Table: *Balamuthia mandrillaris* active compounds identified through dose-response (N=2)			
**Class**	**Compounds**	**qAC$_{50}$'s (µM) ± (SE Log)**	**Proposed Mechanism of Action**
Antineoplastics	MLN-2480	0.04 (0.16)	Raf kinase inhibitor
	Aminothiadiazole	0.21 (0.19)	Inosine monophosphate dehydrogenase inhibitor
	DP-4978	0.25 (0.08)	Raf kinase A, B and C Inhibitors
	10-Deazaaminopterin	0.38 (0.10)	Folate Antagonists
	Tivozanib (ophthalmic)	0.39 (0.04)	Tyrosine Kinase Inhibitors††; VEGFR-1,2,3 (Flt-1,FLK-1/KDR,FLT4) Inhibitors
	Chromomycin A3	0.42 (0.05)	Inhibits GC-rich DNA-binding protein, suppress TNF-alpha-induced fractalkine mRNA expression through NF-kappaB and Sp1
	Dasatinib	0.43 (0.17)	Multi-targeted inhibitor that targets Abl, Src and c-Kit
	Duazomycin	0.47 (0.05)	Glutamine antagonists
	Pelitrexol	0.54 (0.03)	Glycinamide Ribonucleotide Formyltransferase (GARTFase) Inhibitors
	PF-04691502	0.56 (0.06)	Mammalian Target of Rapamycin (mTOR; FRAP1) Inhibitors; Phosphatidylinositol 3-Kinase (PI3K-a, -b, -g, -d) Inhibitors
	Quisinostat	0.71 (0.03)	Histone Deacetylase 1 (HDAC1) Inhibitors
	SU-9516	0.79 (0.24)	Cyclin-dependent kinase 2 inhibitor
	Rebastinib	0.80 (0.04)	Bcr-Abl inhibitor for Abl1(WT) and Abl1(T315I); SRC, LYN, FGR, HCK, KDR, FLT3, and Tie-2, and c-Kit
	Bisnafide mesilate	0.82 (0.15)	DNA synthesis inhibitor; DNA-Intercalating Drugs; RNA synthesis inhibitor;
	SB-2343	0.83 (0.03)	mTOR Complex 1 (mTORC1&2); Phosphatidylinositol 3-Kinase alpha (PI3K-a, -b, -d, -g) Inhibitors;
	AR-42	0.85 (0.03)	Histone Deacetylase 1&2 (HDAC1&2); Protein kinase B inhibitor
	LY-2874455	0.85 (0.06)	FGFR1,2,3&4 Inhibitors;
	NVP-HSP990	1.17 (0.09)	Heat Shock Protein 90 (HSP90) Inhibitors;
	Voxtalisib hydrochloride	1.25 (0.02)	mTOR Complex 1&2 (mTORC1&2) Inhibitors; Phosphatidylinositol 3-Kinase alpha (PI3K-a) Inhibitors
	AZD-8835	1.65 (0.05)	Phosphatidylinositol 3-Kinase (PI3K-a,-d) Inhibitors
	Dabrafenib mesylate	2.08 (0.04)	Raf kinase B Inhibitors
	ES-285	2.69 (0.03)	ACaspase 3&12 Activators; Rho kinase Inhibitor; Sphingosine Kinase 1 (SphK1) Inhibitors
	PF-03814735	2.91 (0.07)	Aurora Kinase Inhibitors

	Omipalisib	3.02 (0.05)	mTOR Complex 1&2 (mTORC1&2) Inhibitors; Phosphatidylinositol 3-Kinase (PI3K-a,-b,-d,-g) Inhibitors
	Milciclib maleate	3.24 (0.03)	CDK1,2,4,&5 Inhibitors; TrKA tyrosine kinase inhibitor
	M3814	4.24 (0.03)	DNA-Dependent Protein Kinase (DNA-PK) Inhibitors
	LY-2457546	4.65 (0.04)	Ephrin (EPH) Inhibitors; Flt3 (FLK2/STK1) Inhibitors; RET Inhibitors; VEGFR-3 (FLT4) Inhibitors
	LTX-315	4.83 (0.01)	Cell membrane structure modulators
Antiseptics	Octenidine	0.46 (0.03)	Membrane integrity inhibitor
	Analog of Decamethoxine	0.99 (0.60)	Acetylcholinesterase Inhibitor
	Alexidine	1.15 (0.04)	Phospholipase Inhibitor
	Decamethoxine	1.16 (0.06)	Acetylcholinesterase Inhibitor
	Mercufenol chloride	1.24 (0.14)	Antiseptic
	Alexidine dihydrochloride	1.39 (0.05)	Phospholipase Inhibitor
	Phenylmercuric borate	1.62 (0.05)	Disinfectant
	Trichlobisonium chloride	2.36 (0.04)	Antiseptic
	Thimerosol	2.90 (0.08)	Glutathione Transferase Inhibitors; Lipoxygenase Inhibitors
Antihyperlipidaemics	Cerivastatin sodium	0.09 (0.02)	3-hydroxy-3-methyl-glutaryl-coenzyme A (HMG-CoA)
	FCE 25390	0.17 (0.10)	Phosphatidylinositol 3-Kinase delta (PI3K-d) Inhibitors
	Bervastatin	0.65 (0.12)	3-hydroxy-3-methyl-glutaryl-coenzyme A (HMG-CoA)
	Pitavastatin calcium	2.25 (0.03)	3-hydroxy-3-methyl-glutaryl-coenzyme A (HMG-CoA)
	NCX-6560	3.98 (0.05)	3-hydroxy-3-methyl-glutaryl-coenzyme A (HMG-CoA)
Antiprotozoals	G-25	0.72 (0.02)	Choline transport
	Artemisinin	1.16 (0.11)	Cytochrome oxidase; Sarco-endoplasmic reticulum calcium ATPase Inhibitor; DNA synthesis inhibitor
	Lauroguadine	1.88 (0.04)	Antiprotozoal
	Diminazene Aceturate	2.08 (0.07)	Angiotensin-I Converting Enzyme-Related Carboxypeptidase (ACE2) Activators; DNA Topoisomerase II Inhibitors
	Stilbamidine	4.49 (0.06)	Antiprotozoal
Antibacterials	Mithramycin A	1.51 (0.04)	Alcohol Dehydrogenase Inhibitors; It binds to DNA and inhibits RNA, DNA, and protein synthesis

	LTX-109	2.68 (0.04)	Cell membrane modulators
	Erythrosine	3.22 (0.07)	Aetiological agent; Antimicrobial
Antivirals	Verdinexor	0.11 (0.29)	Exportin-1 (CRM1, XPO1) Antagonists; Signal Transduction Modulators
	CMX-001	2.81 (0.05)	DNA Polymerase Inhibitors; DNA directed DNA polymerase inhibitor;
Antifungals	Cycloheximide	2.48 (0.04)	Glycogen Synthase Kinase 3 beta (GSK-3beta; tau Protein Kinase I) Inhibitors;
	Gentian violet	2.50 (0.04)	Cyclin-Dependent Kinase Inhibitor 1B (CDKN1B; p27Kip1)
Anti-inflammatories	Paranyline	2.75 (0.03)	Anti-inflammatory, nonsteroidal
	Fingolimod hydrochloride	3.88 (0.02)	Sphingosine Kinase 1 (SphK1) Inhibitors; Lysophospholipid EDG1,3,6&8 (S1P1,S1P3,S1P4&S1P5) Receptor Agonists
Antidementia	EVP-0334	0.21 (0.03)	Histone Deacetylase (HDAC) Inhibitors
Antirheumatic	MX-68	0.35 (0.12)	Dihydrofolate reductase Inhibitor; Thymidylate synthase inhibitor
Antiallergic	GW784568X	0.69 (0.04)	Glucocorticoid Receptor (GR) Agonists
Muscle Relaxant	Hexafluorenium bromide	1.85 (0.02)	Acetylcholinesterase Inhibitor
Diazo dye	Trypan Blue	2.34 (0.06)	Selectively stains connective tissue
Behavioural disorder therapy	GSK-598809	2.46 (0.09)	Dopamine receptor D3 antagonist
Antipsychotic	PF-3463275	4.54 (0.05)	Glycine transporter 1 Inhibitor
